# A Role for the Membrane in Regulating *Chlamydomonas* Flagellar Length

**DOI:** 10.1371/journal.pone.0053366

**Published:** 2013-01-24

**Authors:** William Dentler

**Affiliations:** Department of Molecular Biosciences, University of Kansas, Lawrence, Kansas, United States of America; Emory University School of Medicine, United States of America

## Abstract

Flagellar assembly requires coordination between the assembly of axonemal proteins and the assembly of the flagellar membrane and membrane proteins. Fully grown steady-state *Chlamydomonas* flagella release flagellar vesicles from their tips and failure to resupply membrane should affect flagellar length. To study vesicle release, plasma and flagellar membrane surface proteins were vectorially pulse-labeled and flagella and vesicles were analyzed for biotinylated proteins. Based on the quantity of biotinylated proteins in purified vesicles, steady-state flagella appeared to shed a minimum of 16% of their surface membrane per hour, equivalent to a complete flagellar membrane being released every 6 hrs or less. Brefeldin-A destroyed *Chlamydomonas* Golgi, inhibited the secretory pathway, inhibited flagellar regeneration, and induced full-length flagella to disassemble within 6 hrs, consistent with flagellar disassembly being induced by a failure to resupply membrane. In contrast to membrane lipids, a pool of biotinylatable membrane proteins was identified that was sufficient to resupply flagella as they released vesicles for 6 hrs in the absence of protein synthesis and to support one and nearly two regenerations of flagella following amputation. These studies reveal the importance of the secretory pathway to assemble and maintain full-length flagella.

## Introduction

Cilia and eukaryotic flagella (the terms will be used interchangeably) serve a variety of sensory and motor functions, some of which are critically important for development and maintenance of embryonic and adult tissues. Cilia arose as modifications of the plasma membrane and the membrane evolved to acquire a lipid and protein composition distinct from the plasma membrane [1]–[4]. A variety of structural linkages link the membrane to the microtubules, so the growth and maintenance of the membrane and microtubules must be well coordinated [5]. Microtubule assembly and disassembly occurs at their distal ends [6] and the balance of assembly and disassembly appears to be regulated by numerous factors, including kinesins, plus-end binding proteins [7], [8], protein kinases [9], [10], intraflagellar transport (IFT) [11], [12], signaling pathways linked to cAMP and Ca^2+^ ions [13], [14], tubulin modifications [15], and ubiquitilation [16]. In many eukaryotes, disassembly is required to reuse basal bodies as centrioles [17].

Membrane assembly and the delivery of specific proteins and lipids are becoming increasingly interesting because a major function of ciliary membranes is signal transduction essential for cell-cell interactions and embryogenesis [2], [3]. The ciliary membrane is continuous with the plasma membrane but is separated by proteins that link the basal body/transition region to the membrane [5], [18]–[20]. Evidence for a distinct periciliary domain to which ciliary proteins are targeted has been found in some, but not all cells [21]. Some proteins are only found in flagellar membranes [22], [23], but others selectively move into or out of flagella [18], [19]. Ciliary membranes also differ from the plasma membrane by their enrichment in sterols, sphingolipids and dually acylated proteins that may select and retain flagellar-specific membrane proteins [1]–[3]. Proteins may enter the ciliary compartment by “targeted delivery”, by “diffusion-retention”, or by a combination of these processes [1]–[3]. Targeted delivery to cilia may be mediated by GTPases that recruit BBSome proteins [24].

The Golgi and secretory pathways are important for ciliary assembly. New flagellar membrane is delivered via the secretory pathway either directly, via vesicles that dock at the flagellar base, indirectly, via vesicles that dock at the plasma membrane, or a combination of both. The importance of the secretory pathway is revealed by the inhibition of ciliogenesis by Brefeldin A (BFA) [25], [26], which induces Golgi collapse, and by depletion of the Golgi-associated proteins IFT20 [27], FAPP2 [28], and an AP1-clathrin-adaptor complex [29]. BBSome-associated proteins involved in membrane trafficking also are essential for the assembly of primary and sensory cilia [30]–[32] as is the exocyst complex component Sec10 [33]. Reduction of ceremide synthesis, which may be involved with vesicle fusion or protein targeting, also reduced the number and length of primary cilia [34], [35]. These studies clearly reveal the importance of the secretory pathway for ciliary assembly.

Once assembled, cilia incorporate newly synthesized proteins into their membranes and cytoplasmic structures [36]–[39], indicating that protein turnover occurs in flagella but the fate of the “old” protein is not well understood. Membrane and protein may be recycled, via endocytosis at ciliary bases [40]–[42] or may be released from cells as vesicles are shed from flagellar tips [43]–[Bibr pone.0053366-Brown1] [46]. Perhaps the most dramatic example of membrane shedding from ciliary tips is found in mammalian photoreceptor outer segments, in which the cilia must replace the ∼418,000 rhodopsin molecules and ∼3.2 cm^2^ of membrane that is shed each hour [47].

This study was carried out to examine the role of the membrane in flagellar growth and maintenance in *Chlamydomonas*. Vegetative cells shed vesicles from the flagella into the medium and, based on quantitation of surface-labeled protein, each flagellum shed it's entire membrane during a 6 hr incubation period. Replacement of the lost membrane did not require protein synthesis and new surface-labeled proteins were recruited from a biotinylated pool of plasma membrane proteins. Destruction of the Golgi and secretory system with BFA, however, inhibited flagellar assembly and induced flagellar disassembly, suggesting that failure to resupply the lost flagellar membrane induced flagellar disassembly. IFT was not significantly affected by BFA. These results suggest that the resupply of flagellar membrane lipids is essential both for flagellar assembly and maintenance.

## Results

### Membrane must be supplied to grow and to maintain flagella

**Figure 1:**
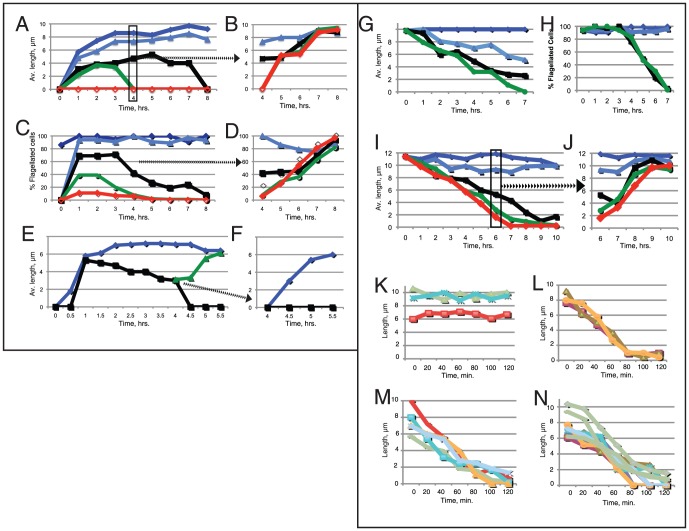
BFA reversibly inhibited flagellar regeneration (A–F) and induced fully grown flagella to disassemble (G–N). A–D: Flagellar lengths on WT cells regenerating with 0 (diamonds), 3.6 µM (triangles), 18 µM (squares), 36 µM (circles), or 54 µM BFA (open diamonds). A: Four hrs after deflagellation, a portion of cells was washed suspended without BFA (B). C–D: percentage of flagellated cells. E, F: Regeneration of deflagellated *pf16* without (diamonds) or with (squares) 18 µM BFA. Flagella regrew to control lengths upon BFA removal (triangles). F: Cells regenerated in BFA for 4 hrs (E) were re-deflagellated and incubated without (diamonds) or with (squares) 18 µM BFA. G, H: Flagellar lengths (G) and percentage of flagellated cells (H) when WT cells were treated with 0–36 µM BFA. I: Flagellar lengths on WT cells treated with 0 µM (diamond), 3.6 µM (triangles), 18 µM (squares), 36 µM (dots), 54 µM (open triangles). BFA. J: Flagellar on cells washed free of BFA at 6 hr (I, box). K–N: To determine if BFA induced cells to deflagellate, individual *pf16* cells were recorded at 1 min intervals without (K) or with 18 µM (L), 36 µM (M), or 54 µM BFA (N). Each curve represents the length of flagella on an individual cell. Videos of representative cells are shown in Supplementary movies S1, S2, S3, S4.

Membrane vesicles (FV) are continually shed from the tips of flagella on *Chlamydomonas* vegetative and gametic cells (Fig. S1) [44]–[46]. One consequence of this shedding is that membrane lipids and proteins must be resupplied to maintain fully-grown flagella. To determine if the membrane can be supplied from a plasma membrane pool or if it needs resupply via the secretory pathway, cells were treated with BFA to disrupt the secretory pathway and the effects of BFA on flagellar assembly and maintenance was examined. 54 µM BFA completely inhibited regrowth after deflagellation and 18–36 µM BFA permitted regrowth but flagella then shortened (Fig. 1A,C). BFA did not induce deflagellation and up to 54 µM BFA was not toxic because flagella regrew upon BFA removal (Fig. 1B,D).

Low concentrations of BFA did not inhibit initial flagellar growth, so it was possible that partially dissociated Golgi provided the required membrane or that growing flagella could draw from a plasma membrane pool. To test this, cells were deflagellated, incubated with 18 µM BFA (Fig. 1E) and were deflagellated a second time and allowed to grow with or without BFA. BFA completely inhibited the second regeneration but, without BFA, flagella regenerated normally (Fig. 1F). Most data presented here were obtained using wild-type (*L8*) *Chlamydomonas* cells but identical results were obtained in preliminary experiments with the temperature-sensitive kinesin-2 mutant *fla10.1*. The paralyzed mutant *pf16* was used to minimize flagellar motility during time-lapse recordings (Fig. 1, supplemental videos) and *pf18* was used to minimize flagellar beating and obtain straight flagella for IFT analysis. Each of these mutants responded the same as WT cells with regard to flagellar length in the presence of BFA. These results suggest that partially dissociated Golgi could support one round of flagellar regeneration but there was insufficient membrane for a second round of growth.

Flagellar shortening induced by BFA suggested that new membrane might be required. To examine shortening, BFA was added to cells with full-length flagella (Fig. 1G–N). At least 95% of the cells contained 2 equal length flagella at the start of the experiment. Low BFA concentrations (2.6 µM) induced some shortening but higher concentrations induced shortening at rates up to 1.6 µm/hr (Fig. 1G). Upon removal of BFA, flagella rapidly regrew to normal lengths (Fig. 1J). Greater than 90% of cells were flagellated 2 hrs after BFA removal and essentially identical results were obtained with *pf18*, *pf16*, *fla10*, and *L8* cells.

The decrease in flagellated cells after 3 hrs in BFA (Fig. 1H) might be due to the difficulty of identifying short flagella in randomly sampled cells or to cells being deflagellated. To confirm that BFA did not induce deflagellation, *pf16* cells were incubated without and with 18–54 µM BFA and images were recorded at 1 min intervals. Time-lapse videos of cells treated with no BFA (Movie S1) or with 18–54 µM BFA (Movies S2, S3, S4) revealed that flagella shortened and cells did not deflagellate. The lengths of flagella on 48 cells were measured and flagellar lengths on representative cells are shown in Fig. 1 K–N. Flagella on control cells did not shorten (Fig. 1K) but cells incubated with 18–54 µM BFA shortened at 3–6 µm/hr (Fig. 1L–N). Shortening rates varied on individual cells but the average shortening rates of cells treated with BFA averaged 3–6 µm/hr, similar to that observed in fixed cells (Fig. 1G–J).

To confirm that BFA destroyed Golgi, BFA-treated cells were fixed and examined by electron microscopy (Fig. S2). Cells incubated with 36 µM BFA contained no Golgi but Golgi reappeared when cells were fixed 30 min after BFA removal. Cells treated with 1.4 or 3.8 µM BFA lacked normal Golgi but contained possible Golgi remnants.

### Membrane released at flagellar tips is replaced by a pool of membrane and membrane proteins

**Figure 2:**
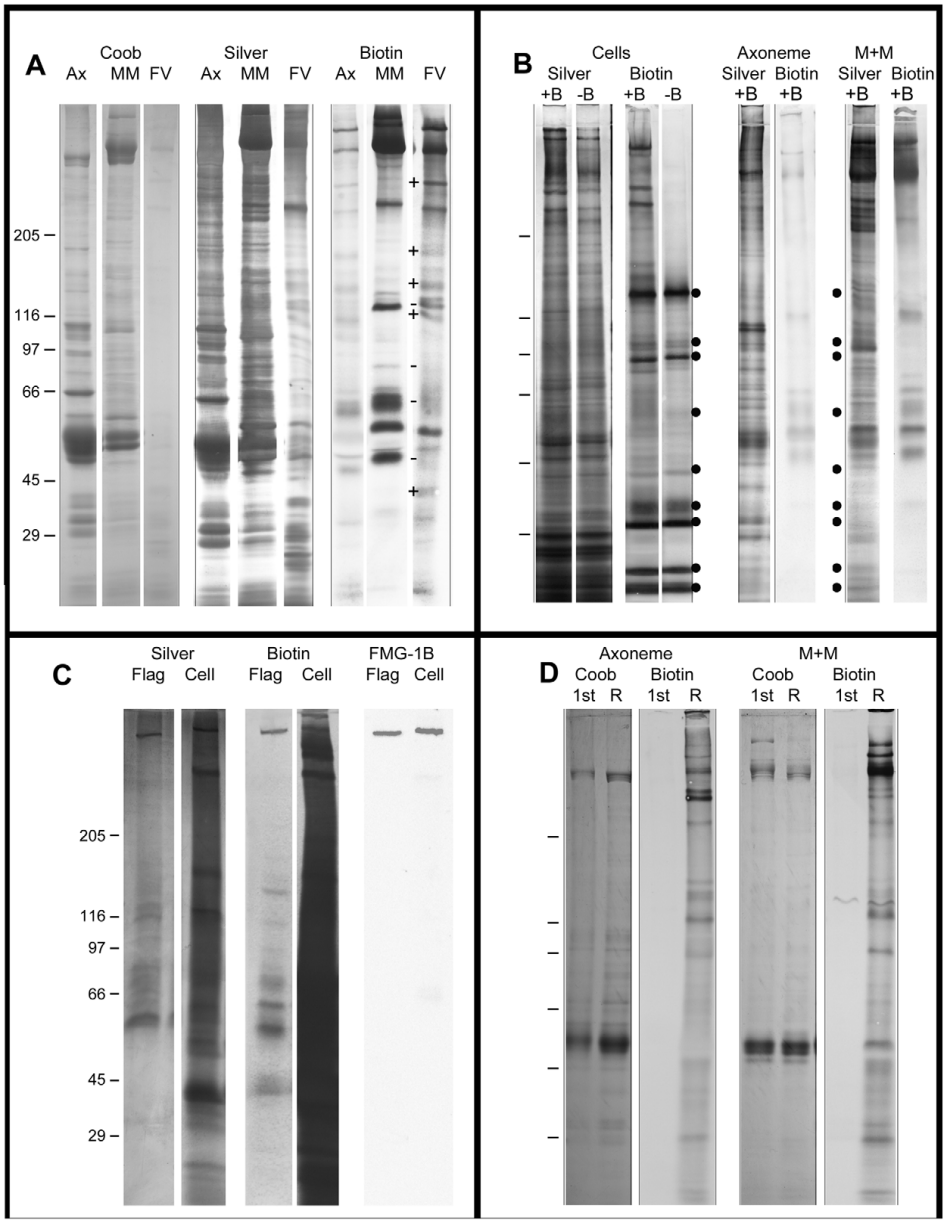
Analysis of control and biotinylated *Chlamydomonas* cells, purified flagellar vesicles shed into the medium, and the detergent-insoluble axonemes and detergent-solubilized membrane+matrix from purified flagella. A. Composition of FV compared with axonemes and membrane+matrix. “+” and “−” refer to the relative quantity of biotinylated proteins in the membrane+matrix (MM) and the flagellar vesicles (FV). B. Identification of endogenously biotinylated proteins in cells but not flagella. Biotinylated (+B) and non-biotinylated (−B) cells were deflagellated. Black dots mark endogenously biotinylated bands. These were not found in flagellar fractions or in FV (compare B with FV in A). C. Biotinylated proteins from flagella and deflagellated cells were affinity-purified by avidin chromatography and separated by SDS-PAGE. Identically loaded blots were stained with AP-streptavidin (Biotin) or antibodies against FMG-1B. D. Flagella isolated from non-biotinylated cells (1^st^) contained no endogenously biotinylated bands. Deflagellated cells were biotinylated and allowed to regenerate flagella. Regenerated flagella contained the same biotinylated proteins seen when flagellated cells were biotinylated.

By disrupting the secretory pathway, BFA likely prevents the delivery of membrane and, possibly, proteins required for flagellar assembly. This is consistent with studies of Rab8 requirements for the formation of primary cilia [30]. Flagellar shortening induced by BFA, however, suggested that flagellar membrane must be replenished as membrane is recycled, via endocytosis, or shed as flagellar vesicles. To study the release of flagellar membranes, *Chlamydomonas* surface-exposed proteins were pulse-labeled with biotin using a vectorial label that cannot penetrate membranes (Fig. 2D Biotin 1^st^). Biotinylation also focused attention on proteins that specifically were attached to the membrane and not those that were contained within the flagella or flagellar vesicle matrix.

For each experiment, cells were pulse-labeled with the biotinylation reagent for 10 minutes and washed twice in M medium to remove free reagent. Vesicles, cells, or isolated flagella fractionated with Nonidet P-40 were fractionated by SDS-PAGE and identically loaded gels were stained for protein or were blotted to nitrocellulose and stained with alkaline phosphatase-streptavidin to identify biotinylated proteins. Most biotinylated flagellar polypeptides were solubilized by NP-40 and consisted of 6 HMW bands (275–313-kDa), and bands at 208, 115, 63, 53, and 50-kDa (Fig. 2A). The high molecular weight doublet band likely is the major flagellar glycoprotein, FMG-1b and the 208 kDa band is likely mastigonemes, attached to the surface of *Chlamydomonas* flagella.

To identify proteins associated with the shed vesicles, biotinylated cells were incubated for 5–6 hrs and vesicles were isolated from the medium. At the end of the incubation period, more than 95% of the cells were motile and flagellated and the average flagellar length was 9.5±1.4 µm, slightly longer than flagella on cells at the start of the incubation. The vesicles contain a minimum of 20 proteins, at least 15 of which are biotinylated and, therefore, exposed to the flagellar surface (Fig. 2A). The composition of flagellar membrane+matrix and vesicles are not identical. Flagellar vesicles contained most of the biotinylated proteins in the membrane+matrix fractions but some membrane+matrix proteins were enriched (Fig. 2A “+”) and some reduced (Fig. 2A “−”) in the vesicle fraction. Coomassie blue and silver stained flagellar vesicle proteins differ from the axonemes, confirming that vesicles fraction is not contaminated by flagella. Another concern was that the vesicle fraction was contaminated with cell debris. To assay for cells or debris, western blots of cells that had not been treated with the biotinylation reagent were stained with AP-streptavidin to reveal endogenously biotinylated polypeptides, presumably carboxylases [48], protein ligases [49], amidolyases [50] and possible cell wall components (Fig. 2B). These endogenously biotinylated proteins served as sensitive markers for cells or cell fragments. The vesicle fractions did not contain these endogenously biotinylated proteins (Fig. 2b), which confirmed that the vesicles were shed from flagella and were not produced from cells or cell debris.

Although the *Chlamydomonas* cell wall is permeable to proteins as large as 100 kDa [51], [52], it was important to determine if the 557 Da biotinylation reagent could penetrate the cell wall and label plasma membrane proteins. Flagellated cells were biotinylated, washed, deflagellated, and biotinylated proteins were purified from purified flagella and deflagellated cell bodies by streptavidin affinity-chromatography. The major biotinylated flagellar proteins were purified from flagella (Fig. 2C Biotin Flag) and numerous biotinylated proteins were purified from the cell bodies (Fig. 2C Biotin Cell). The major flagellar glycoprotein, FMG-1B [53], was identified in both flagellar and cell body fractions using an antibody to FMG-1B (Fig. 2C FMG-1B), which confirmed that the reagent was freely accessible to plasma membrane surface-exposed proteins.

If flagellar shortening is partially due to the BFA-mediated inhibition of membrane resupply via the secretory pathway, the amount of membrane shed during a 5–6 hr period should be a substantial proportion of the flagellum. To estimate of the quantity of membrane shed by flagella, biotinylated cells were incubated for 5–6 hours and biotinylated proteins in and the quantity of biotinylated flagellar vesicle proteins was compared with the quantity of biotinylated proteins in purified flagella. Based on stoichiometric loadings, the percentage of biotinylated flagellar protein in the vesicles relative to flagella was determined (Table 1, Fig. S3). The averages of 5 different experiments revealed that, on average, the flagellar vesicle fraction contained 100% of the biotinylated protein found in isolated flagella. Individual bands significantly differed in the quantity of shed protein. Some contained 60–80% of the flagellar protein while others contained greater than 150% of the flagellar protein. The amounts varied in different experiments but in no experiment did the vesicles contain less than 40% of the biotinylated protein present in the flagellar membrane+matrix. These results indicate that, over a 6 hr period, flagella shed at least 100% of the biotinylated proteins in flagellar vesicles, or approximately 16% of flagellar surface proteins/hour. Given the loss of likely loss of flagellar vesicles during purification, this may be an underestimate of the amount of membrane shed.

**Table 1:** Percentage of biotinylated flagellar protein bands recovered in flagellar vesicle fractions after 6 hr incubation.

		Percent of biotinylated protein in flagellar vesicles				
Band	Av%	Expt 1	Expt 2	Expt 3	Expt 4	Expt 5
1 (1 and 2 = FMG-1b doublet)	80±60	40	30	40	180	90
2	50±20	40	30	20	90	60
3 (mastigoneme)	130±90	160	40	50	130	280
4	90±40	80	60	40	110	150
5	270±60		340	260	220	
6	180±170	70	30	380	70	370
7	70±8	70	70	60	80	80
8	60±40	40	50	30	70	130
Av % total flagellar biotinylated protein recovered in flagellar vesicles	120±70	70	80	110	120	160

**Figure 3:**
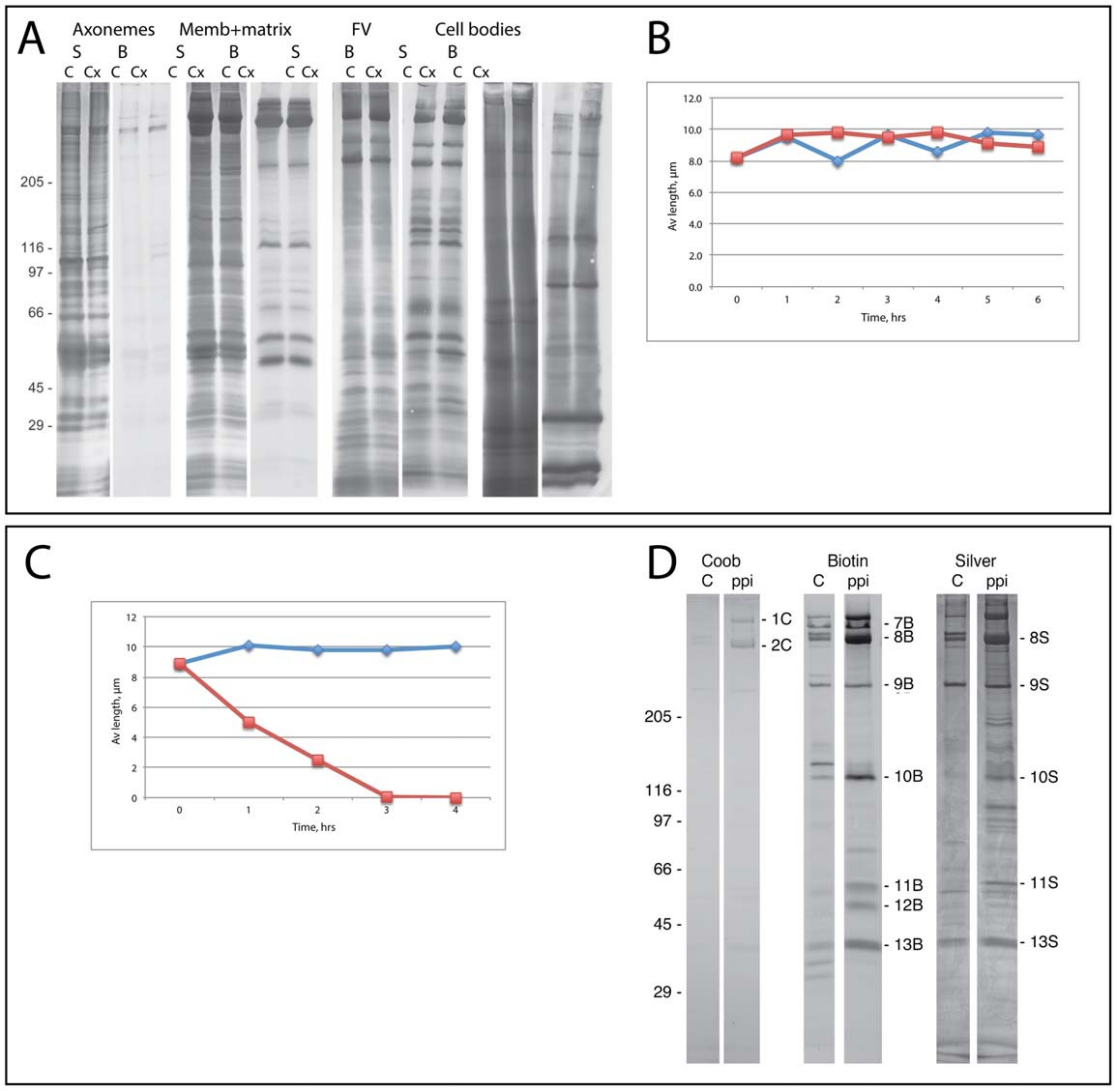
Flagellar vesicles continued to be shed and surface proteins replaced as cells were incubated with cycloheximide, to inhibit protein synthesis while maintaining full-length flagella (A, B). Sodium pyrophosphate induced flagellar shortening and increased the release of flagellar vesicles (C, D). A: Biotinylated cells were incubated without (C) or with (Cx) 10 µg/ml cycloheximide for 6 hrs and then were pelleted, deflagellated, and flagella were extracted with NP-40 to produce axonemes and membrane+matrix. Shed flagellar vesicles (FV) were purified from the medium and cell bodies were sedimented after deflagellation. Identical volumes of axonemes, membrane+matrix, and FV were stained for total protein (S) or biotinylated protein (B). B: Aliquots of cells were fixed during the 6 hr incubation and flagellar lengths were measured on control cells (diamonds) or cycloheximide-treated cells (squares). Cycloheximide did not induce flagellar length changes. C: Cells with full-length flagella (diamonds) maintained full length while 20 mM sodium pyrophosphate induced flagella to shorten (squares). D: Equal volumes of biotinylated cells were incubated without (C) or with (ppi) pyrophosphate for 4 hrs as flagella on pyrophosphate-treated cells resorbed. Shed flagellar vesicles were purified, suspended in equal volumes of buffer, fractionated by SDS-PAGE, and stained for total protein (Coob, Silver) or for biotinylated proteins (Biotin). Densitometric analysis of individual bands, identical in MW to those analyzed in regenerating flagella (Fig. 4) is presented in Table 2.

Densitometric analysis of biotinylated bands in detergent extracts (membrane+matrix) of purified flagella and released flagellar vesicles. Biotinylated cells were incubated for 6 hrs. Cells were pelleted, deflagellated, and flagella were purified and extracted with NP-40. Vesicles were purified from the culture medium. Membrane+matrix and vesicle polypeptides were fractionated by SDS page and biotinylated proteins were stained on western blots. The densities of eight bands (Fig. S3) were measured and the proportion of polypeptides in the vesicle fraction compared to that in each membrane+matrix was determined based on the percentage of the sample loaded on the gels. Measurements from 5 different experiments and the average recovered protein for each of the bands are shown. The amount of FV protein recovered varied with different bands. A value of 100% indicates that the FV contains 100% of the biotinylated protein present in each flagellum. During the incubation, flagella maintain their quantities of biotinylated protein by drawing on the plasma membrane pool (see Fig. 3A).

Steady-state flagellar membranes rapidly incorporate newly synthesized proteins [36]–[38] but inhibition of protein synthesis for 8–12 hours does not induce flagellar shortening [36], [37]. This suggests that a pool of flagellar proteins is stored somewhere in the cell or that turnover does not occur without protein synthesis. However, new protein must be added to the flagellum to replace the protein and membrane lost as flagellar vesicles are released. To examine protein replacement, biotinylated cells were divided into equal volumes and incubated for 6 hrs with or without the protein synthesis inhibitor cycloheximide (CX). It was predicted that incubation in CX would reduce the pool of flagellar surface proteins within the plasma membrane or in the flagella or that CX would reduce the rate of release of vesicles. However, CX treatment did not affect the quantity or density of biotinylated bands present on axonemes, membrane+matrix, flagellar vesicles, or cell bodies nor did it induce flagellar shortening (Fig. 3A,B). Therefore, the plasma membrane must contain a pool of proteins sufficient to resupply flagellar membrane proteins for at least 6 hours as they are turned over by the cell or released as flagellar vesicles.

If steady-state flagella continually release significant quantities of membrane, do they release vesicles as flagella shorten? To examine this, flagella were induced to shorten with Na^+^-pyrophosphate [54]. When examined by electron microscopy, large vesicles were released from the flagellar tips (Fig. S1), suggesting that membrane may be released into the medium rather than being returned to the cell body when flagella rapidly shorten. To examine this more rigorously, biotinylated cells were divided into two equal aliquots, one of which was incubated in M medium (control cells) and the other in M plus 20 mM pyrophosphate. Cells were incubated until flagella on pyrophosphate-treated cells disassembled (Fig. 3C). The shed flagellar vesicles were purified and equal volumes of the vesicle pellets were fractionated by SDS-PAGE and examined for total protein (Coob, Silver) and for biotinylated proteins (Biotin). The pyrophosphate-treated cells released more than 3 times the amount of biotinylated vesicle protein than was found in control cells (Fig. 3D, Table 2), suggesting that flagella shed membrane as they disassembled.

**Table 2:** Densitometric analysis of shed FM from control and sodium pyrophosphate-treated cells.

Coomassie blue stained bands	Density X Control	Biotinylated bands	Density X Control	Coob+Silver stained bands	Density X control
1C	3.3	7B	2.2		
2C	3.0	8B	2.7	8S	1.4
		9B	2	9S	1.6
		10B	5.3	10S	6.6
		11B	4.4	11S	2.3
		12B	5.4	-	-
		13B	4.4	13S	1.7
Av increase	3.2		3.8		2.7

Stained bands from gels shown in Fig. 3D were analyzed with ImageJ and the density of individual flagellar vesicle isolated from pyrophosphate-treated cells is expressed as a multiple of the density of the same bands released by control cells. Bands are selected and numbered as described in Figure 3B.

### Surface-exposed flagellar membrane proteins are replaced from a plasma membrane protein pool

The release of a substantial portion of the flagellar membrane by nongrowing flagella indicates that the flagellar membrane proteins must be replenished either by protein synthesis or by drawing proteins from the plasma membrane. To determine if the plasma membrane is a source of flagellar surface proteins, cells were deflagellated and plasma membrane proteins were biotinylated in the presence of colchicine to prevent flagellar regrowth. Cells were washed free of biotin and colchicine, were allowed to regenerate flagella, and were deflagellated. Flagella isolated before biotinylation and flagella isolated after regeneration from biotinylated cells were analyzed for the presence of biotinylated proteins. Flagella isolated from cells before treatment with the biotinylation reagent lacked biotinylated proteins (Fig. 2D, 1^st^) but flagella regenerated from biotinylated cells contained a complement of biotinylated polypeptides (Fig. 2D R) similar to that in flagella that had been biotinylated on flagellated cells (Fig. 2A). Therefore, growing flagella can recruit labeled proteins directly from the plasma membrane.

**Figure 4:**
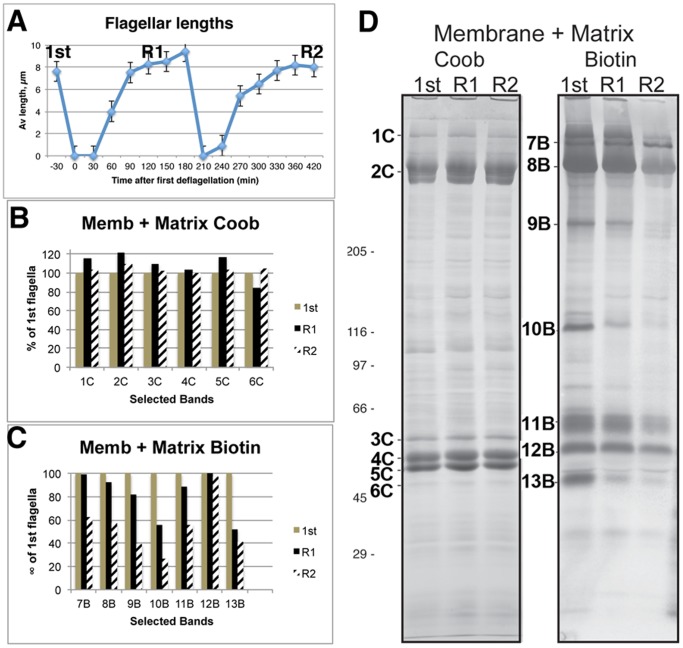
A pool of flagellar surface proteins on the plasma membrane can supply regenerating flagella. Cells were biotinylated and flagella were isolated from the freshly biotinylated cells (1^st^). Cells regenerated flagella, deflagellated (R1), and regenerated flagella a second time (R2). A: Flagellar lengths before the first deflagellation (1^st^), first (R1) and second (R2) regeneration. B–D: Membrane+Matrix stained with CB or blotted and stained with AP-streptavidin (Biotin). Density of selected bands stained with CB (1C–6C) or AP-streptavidin (6B–13B) presented as a percentage of the density of the bands in flagella isolated immediately after biotinylation (1^st^). Coomassie blue stained proteins in each fraction were nearly identical (B) but the quantity of biotinylated protein decreased with each regeneration (C), indicating that the pool of biotinylated protein was gradually depleted as flagella grew.

To estimate the size of the flagellar surface protein pool on the plasma membrane, biotinylated cells were subjected to two rounds of deflagellation and flagellar regeneration (Fig. 4A). Flagella isolated from freshly biotinylated cells (1^st^, Fig. 4 B–D) were compared with flagella isolated after one (R1) and two (R2) regenerations. Biotinylated proteins were in all three flagellar samples, confirming that a pool of flagellar proteins on the plasma membrane can resupply nearly two complete rounds of flagellar growth. The intensity of biotinylated protein bands varied, indicating differences in the size of the plasma membrane protein pool. Based on band density, the size of the plasma membrane pool of polypeptides 7B, 8B, 9B, 11B, and 12B was sufficient to provide 80% of the protein required for the first regeneration and 40–60% of that required for a second regeneration (Fig. 4C,D) but the pool of Band 12B was sufficient to supply most or all of the protein required for a second regeneration. The quantity of biotinylated protein shown here is a low estimate of the plasma membrane pool because the Membrane+Matrix proteins were released after one extraction of flagella with NP-40 and a second extraction of the flagella released 10–20% more protein. These results confirm that regenerating flagella draw from a pool of surface-exposed (biotinylated) plasma membrane proteins.

Because intraflagellar transport (IFT) may be related to flagellar growth and maintenance, IFT was recorded and kymographs were analyzed to determine IFT particle frequency and rates. Compared to control cells, 3.6 µM BFA reduced the number of IFT tracks by 5–10% and 36 µM BFA IFT tracks to 60–70% of controls. The ratios of anterograde: retrograde tracks/second and IFT particle rates were not affected by BFA. IFT was never reduced to less than 60% of that in controls in the highest concentrations of BFA tested. BFA also inhibited flagellar assembly and increased shortening rates in *pf18* cells. IFT in control (no BFA) and in *pf18* cells+36 µM BFA were compared and I found no differences in IFT frequency or anterograde IFT rates (417 tracks measured), a 20% decrease in retrograde IFT rate but not frequency (365 tracks measured), and the size of anterograde and retrograde particles (judged by track width) were identical (448 particles measured). Taken together, these data indicate that BFA-induced flagellar shortening is unlikely to be caused by attenuation of IFT.

## Discussion

The ciliary membrane forms the ciliary compartment and displays cell-surface proteins essential for sensory reception and cell adhesion [2], [3], [21]. The membrane is supported by the microtubular axoneme and in most cells the growth and shortening of axonemes and membranes is closely coordinated. Currently, we have little understanding about the crosstalk between the membrane and the axoneme and any contribution that the membrane may play in ciliary length regulation.

To assemble cilia, new membrane and proteins must be delivered either from the Golgi or from the plasma membrane [24]–[27]. Newly synthesized proteins are rapidly incorporated into the flagellar membrane+matrix [36]–[39] but inhibition of protein synthesis does not affect flagellar length or function (this report) [13], [36], [37] which suggests that newly synthesized proteins add to a pool of proteins sufficient to maintain flagella without protein synthesis. What happens to the “old” flagellar proteins? Axonemal components can be recycled to form new flagella [55] and some cilia may recycle membrane by endocytosis [42]. However, algal and mammalian cilia also shed membrane from their tips [1], [44]–[46] and data reported here reveal that each flagellum on vegetative *Chlamydomonas* cell sheds a minimum of 16% of its flagellar surface each hour, a rate that would replace the entire flagellar membrane every 6 hours. Membrane shedding also increased when flagellar disassembly was induced with sodium pyrophosphate. Therefore, similar to vertebrate photoreceptors [47], *Chlamydomonas* flagella continually shed membranes at their distal tips and, as flagella shorten, membrane is likely to be released rather than recycled via endocytosis. This continual release of membrane may explain pulse-labeling experiments in which labeled proteins are rapidly incorporated into flagellar membranes and then rapidly disappear [37]. If the membrane proteins were recycled by endocytosis one would expect them to reappear over time but they would not reappear if they were released with vesicles shed into the medium.

The membranes and membrane proteins that are lost with the shed vesicles must be replaced to maintain flagella. Surface-exposed membrane proteins were replenished from a plasma membrane pool that may have been transported laterally from the plasma membrane into the flagellum [18], [23], [24], [25] or may be targeted by endocytic recycling. However, the net replenishment of flagellar membrane is most likely due to the delivery of new membrane via the Golgi to the flagellar base [2], [3], [57] or to the plasma membrane. Disruption of the Golgi and secretory system with BFA, would inhibit the ability of the cell to provide new membrane and, as membrane is shed from flagellar tips, the lack of membrane resupply may stimulate the flagellar disassembly reported here.

Inhibition of secretion should affect both protein and membrane delivery to flagella. CX, a protein synthesis inhibitor, inhibits protein synthesis and prevents *Chlamydomonas* cells from regenerating more than half-length flagella [58] but treatment of cells with full-grown flagella with CX for up to 10 hours does not induce changes in flagellar length or motility. By contrast, BFA treatment inhibits flagellar growth and induces flagellar disassembly. Therefore, delivery of membrane to the cell surface is essential to grow and maintain *Chlamydomonas* flagella but these cells contain a pool of flagellar proteins sufficient for limited flagellar growth and to maintain existing flagella.

*Chlamydomonas* flagella can draw from a plasma membrane pool of flagellar surface proteins that is sufficient to replace proteins lost as flagellar vesicles are shed. Based on the quantity of flagellar membrane shed during six hours, the protein pool must be sufficient to allow each flagellum to completely replace its membrane. This is consistent with the observations that biotinylated proteins on the plasma membrane were sufficient to resupply *Chlamydomonas* cells for one and most of a second complete regeneration of flagella. The plasma membrane, therefore, contains a pool of surface-exposed (biotinylatable) proteins that can resupply proteins lost as vesicles shed and to completely resupply regenerating flagella.

The location of the flagellar protein pool on the plasma membrane is not known. In some cells, ciliary membranes and proteins may be specifically transported from the Golgi to the flagellar pocket, a specialized region at the base of the flagellum [2], [3], [57] and the concentration of extraflagellar mastigonemes near flagellar bases [59] supports that model for *Chlamydomonas* even though *Chlamydomonas* lacks a distinct pocket. However, other studies show that *Chlamydomonas* sexual agglutinins can travel in and out of the flagellar membrane, presumably by diffusion across the transition zone [18]. The ability of flagella to incorporate biotinylated proteins through two cycles of flagellar regeneration suggests that the pool of flagellar surface proteins is not concentrated at flagellar bases but, rather, is distributed on the cell surface. Secretory vesicle membranes may be delivered near flagellar bases and may or may not remain in the vicinity of the flagellar base or pocket. Since inhibition of vesicle trafficking but not inhibition of protein synthesis inhibits flagellar assembly and induces flagellar disassembly, the transport and fusion of vesicles near flagellar bases may not require that the vesicles contain flagellar membrane proteins.

Electron microscopy confirmed that BFA destroyed *Chlamydomonas* Golgi but might BFA target other coated vesicle- mediated membrane trafficking? BFA is a specific inhibitor of Sec7-GEFs that activate Arf1p [60]–[63], collapses Golgi, and inhibits secretion and endosomal membrane recycling in plant and animal cells [60]–[65]. *Chlamydomonas* contains two putative Sec7-domain proteins that are predicted to code for Arf-GEFs, so the BFA targets are present. BFA also blocks endosomal trafficking in some cells [61], so it is possible that BFA may induce flagellar shortening by blocking recycling of previously assembled tubulin or other flagellar proteins. If the BFA effect were due to an inhibition of endocytosis, one might expect to observe an increase in flagellar membrane, similar to that observed when endocytosis was inhibited by gene knock-downs in *C. elegans* [42], but this was not observed here. Other effects of BFA may include increasing HSP70 [26] and affecting Ca^2+^ homeostasis [66], but changes in Ca^2+^ levels can affect *Chlamydomonas* motility and induce deflagellation [67] but even high BFA concentrations did not induce deflagellation or alter motility, so our interpretation is that the major effect of BFA is to inhibit the secretory pathway-mediated delivery of membrane to the cell surface.

The role of membrane trafficking for the assembly of primary cilia has been studied in a variety of cells. Primary cilia require BBSome-associated Rab8 [30], [31], [68], [69], Rabin8 (a Rab8-GEF) [32], [68], and Rab11 [32], [69], each of which is essential for membrane trafficking [69], [70]. A putative Rab-related GTPase is present in *Chlamydomonas* but its relationship to Rab8 is not known and no Rabin8-related protein appears to be present. If the *Chlamydomonas* Rab8 does participate in membrane delivery to the plasma membrane or flagellum, it is unlikely to be affected by BFA, because Rabin8 lacks the Sec7-domain to which BFA binds. In addition to BBSomes, exocyst components, associated with secretory vesicle trafficking to membrane, also are required for primary ciliogenesis [71], [72], but exocyst components in *Chlamydomonas* have not been characterized.

How does membrane trafficking regulate flagellar growth and maintenance? One possibility is that the axoneme is sensitive to membrane tension. The flagellar membrane is linked to the distal tips of flagellar doublet and central microtubules by specialized capping structures [5], [73]–[Bibr pone.0053366-Dentler4] [75] and that the tips are the sites of microtubule growth [6], kinesin 13-induced disassembly [8], [10], vesicle budding [46], [46], and IFT remodeling [12], [76]. Flagellar membrane shedding without replacement via the secretory pathway may increase tension on microtubule tips and stimulate capping structures to alter the dynamics of microtubule assembly and disassembly. Consistent with this are observations that microtubule dynamics in vitro are sensitive to membrane tension [77].

A second possibility is that IFT or BBSome trafficking between the Golgi and cell surface might regulate flagellar assembly and maintenance. Membrane-associated BBSomes or IFT particles might be “counted” at flagellar bases and, when BFA blocks the production of new secretory vesicles, the particles might become “stranded” in the cytoplasm. This could be similar to IFT trafficking models that have been proposed to occur within the flagellum [11]. Finally, membrane trafficking and recycling may be required to reposition ciliary membrane components at the proper position on the cell surface [56].

Regardless of the mechanism(s) by which ciliary assembly is regulated, flagellar length is regulated by a balance of protein addition and removal from the axoneme coordinated with a balance of membrane addition and loss, by shedding or endocytosis, from the flagellar surface. We have much to learn about ciliary and flagellar membrane dynamics and the results reported here suggest that that flagellar membranes can play an essential role in flagellar assembly, maintenance and disassembly that warrants further exploration.

## Materials and Methods

### Cell culture, deflagellation, and regeneration

Light-synchronized *Chlamydomonas* cells were cultured as described [13]. To isolate flagella, 4–16 liter cultures were harvested with a Pelicon (Millipore Corp, www.millipore.com), concentrated by centrifugation (3 min at 1,500×G) and suspended in M medium or, for biotinylation, in HM (10 mM HEPES, 5 mM MgSO_4_, pH 7.2). Flagella were amputated by pH shock [78].

For regeneration, deflagellated cells were centrifuged (1,100×G, 3 min.), suspended in M, and incubated with aeration in light. Flagella were isolated from pH-shocked cells by centrifugation (10 min, 706×G). The supernatant was layered over 25% sucrose in TCWB (50 mM Tris, pH 7.4, 3 mM MgSO_4_, 0.1 mM EGTA, 0.25 M sucrose) and centrifuged 10 min, 1622×G, in a swinging bucket rotor. Flagella collected from the interface were pelleted (15 min, 11,290×G), suspended in TCWB and pelleted. Washed flagella were suspended in TCWB, made 1% in Nonidet P-40 (NP-40, Sigma Corp, St Louis, MO), incubated 10 min, 4°C, and pelleted (11,290×G 20 min). The supernatant (Membrane+Matrix (M+M) 1) was saved and the pellet was extracted a second time with 1% NP-40, and centrifuged (11,290×G, 20 min) to produce M+M 2 and axonemes.

### Microscopy

For flagellar length studies, cells were incubated in 6-well dishes. Na^+^-pyrophosphate was prepared as 100 mM stocks in M, pH 7.0 and BFA (Sigma Chemical Corp. #B6542) was prepared as a 36 mM solution in 100% ethanol. Flagella were viewed with phase contrast optics and a Zeiss (Carl Zeiss, www.zeiss.com) WL microscope. Images were captured using a Dage camera (Dage-MTI, Michigan City, IN) and a Macintosh G4 computer with a Scion (Scion, Frederick, MD) frame grabber. Flagella were measured with Image J (http://rsb.info.nih.gov/ij/) and analyzed using Excel (Microsoft Corp, Redmond WA). Only flagellated cells were measured. For clarity, standard errors are not shown, but averaged ±0.9–1 µM. 30–90 flagella were measured for each data point.

For continuous observation and IFT recording, *pf16* cells were chosen because they were paralyzed and populations of the light-synchronized cells had uniform length flagella. Cells were applied to cleaned microscope slides with a drop of M medium or M medium+the appropriate concentration of BFA prepared in 2.5% low EEO agar. Cells were recorded at 1 min intervals for 120 min with low illumination and recorded with a Zeiss WL microscope with a UV filter and 40×/0.75 or 63×/1.4 phase contrast lenses [12], [79].

For electron microscopy, cells were fixed and processed for thin sectioning or negative staining. Samples were viewed and photographed using a JOEL 1200EXII (JEOL.com) or a Technai F21 XT (www.FEI.com) transmission electron microscope.

### Flagellar vesicle isolation

Sixteen liters of cells were harvested and incubated in 600 ml of fresh M for 4–7 hrs with aeration. Cells were pelleted (1,500×G RT) and the supernatant centrifuged at (10,000×G 10 min RT) to remove additional cells. This supernatant was centrifuged (125,171×G, 60 min, 4°C) and membrane pellets were suspended in TCWB, layered over 25% sucrose in TCWB, and centrifuged (10 min 17.130×G, 4°C) in a swinging bucket rotor. The supernatant and interface were collected and centrifuged (31,360×G, 30 min) to pellet vesicles. Samples were frozen at −20°.

### Biotinylation

Cells were centrifuged (1,500×g, 3 min), suspended in 400 ml of HM, and biotinylated with 20 µg/ml EZ Link Sulfo-NHS-LC Biotin (Pierce Thermo Scientific) for 10 min [80]. Cells were diluted with fresh M, pelleted (1,500×G, 3 min), resuspended and washed twice in M medium. Deflagellated cells were biotinylated in HM with 3 mg/ml colchicine. During the final wash, colchicine was washed out and cells were allowed to regenerate flagella.

### Affinity purification

Cells were biotinylated, washed, and deflagellated. Deflagellated cells and flagella were suspended in TCWB and incubated 20 min, 4°C, with 1% sodium dodecyl sulfate (SDS), and centrifuged (25 min, 40,000×G) to pellet SDS-insoluble material. Supernatants were applied to Promega SoftLink Soft Release Avidin Resin in 0.8 ml columns (Pierce-Thermo Scientific) pre-equilibrated with 5 mM biotin and TCWB. Columns were washed with TCWB and proteins eluted with 10 mM biotin in TCWB. Each fraction was divided into three identical samples and polypeptides were separated by SDS-PAGE.

### SDS-PAGE

Polypeptides were separated on 4–16% acrylamide gels, stained with Coomassie blue and, subsequently, silver. Identically loaded gels were blotted to nitrocellulose and stained with alkaline phosphatase-streptavidin [80] or with a mouse monoclonal FMG-1b antibody (provided by Dr. Robert Bloodgood, University of Virginia). The antibody was visualized with HRP-anti-mouse antibody (Dr. Yoshi Azuma, University of Kansas) and a SuperSignalWst Pico Chemiluminescence kit (Pierce Thermo Scientific) and a Kodak 4000R imager.

## Supporting Information

Figure S1 **Vesicles released from the tips of fully-grown flagella (A–D) can be purified from the medium (E, F).** A, B: Flagellar membrane vesicles (FV) are shed from the tips of *Chlamydomonas* vegetative and gametic cells. Vesicles (A, arrowhead) release near or to one side of the central microtubule caps on WT flagella, which contain central microtubules, but from the tips D, arrowhead) of *pf18* flagella, which lack central microtubules and caps. B, C: Larger FV released with 20 mM Na^+^-pyrophosphate. The end of the axoneme is indicated by the arrow. D: FV (arrows) releasing from *pf18* flagella that lack central microtubules. E, F: Shed flagellar vesicles (FV) were purified from the medium in which cells were incubated for 6–8 hours. These vesicles varied in diameter: 78% of vesicles released from WT cells (E) were 20–50 nm in diameter and the rest were larger; 73% of vesicles released from *pf18* were 10–30 nm in diameter. Larger vesicles were produced when flagellar disassembly was induced with sodium pyrophosphate (F). Vesicle diameters range from 60–200 nm diameter and have varied shapes. Bars = 200 nm
(TIF)

Figure S2 **BFA reversibly destroys visible Golgi in** ***Chlamydomonas*** **cells.** To confirm that BFA destroyed Golgi, cells were incubated with 0.4–54 µM BFA for 3 hours and then fixed for TEM. Cross-sections of cells that contained the nucleus and basal bodies were analyzed for the presence or absence of Golgi. In 51 sections of control cells, 42 (82%) of cells had one or more Golgi (A). Analysis of 51 sections of cells treated with 36 µM BFA (B) revealed no cells with Golgi; a repeat of this experiment found one possible Golgi in more than 200 images of the cross-sectioned cells. When cells were washed out of BFA and fixed after 30 min., analysis of 58 cross-sections revealed that 79% of the cells had one or more normal-appearing Golgi (C). With lower BFA, normal Golgi stacks were never observed, but possible Golgi remnants were found in cells treated with 1.4 µM (D) or 2.8 µM BFA (E, F). Flagella in BFA-treated cells looked normal and contained both IFT particles (G) and the distal tips of the A tubules attached to the membrane by distal filaments (H). Therefore, Golgi were absent in BFA-treated cells in which flagellar assembly is partially (3.6 µM) or completely (36 µM) inhibited and in which flagella shorten. Bars = 0.1 µm.
(TIF)

Figure S3 **Western blots of detergent-solubilized flagellar proteins (MM) and released flagellar membranes (FV) stained with AP-streptavidin to reveal biotinylated proteins.** This is one of 5 gels that were analyzed for data presented in Table 1.
(TIF)

Movie S1 **Control** ***pf16*** **cells were recorded at 1 min intervals for 120 min.** Flagella maintained constant lengths throughout the recording period.
(MOV)

Movie S2 ***pf16*** **cells were incubated with 18 µM BFA and were recorded at 1 min intervals for 120 min, as flagella shortened.**
(MOV)

Movie S3 ***pf16*** **cells were incubated with 36 µM BFA and were recorded at 1 min intervals for 120 min, as flagella shortened.**
(MOV)

Movie S4 ***pf16*** **cells were incubated with 54 µM BFA and were recorded at 1 min intervals for 120 min, as flagella shortened.** Even at this high concentration of drug, flagella shortened but cells did not deflagellate.
(MOV)
