# Supplementary figures and images for: A Role for the Membrane in Regulating Chlamydomonas Flagellar Length
Source: PLoS One. 2013 Jan 24;8(1):e53366. doi: 10.1371/journal.pone.0053366 (PMC3554728; doi:10.1371/journal.pone.0053366)

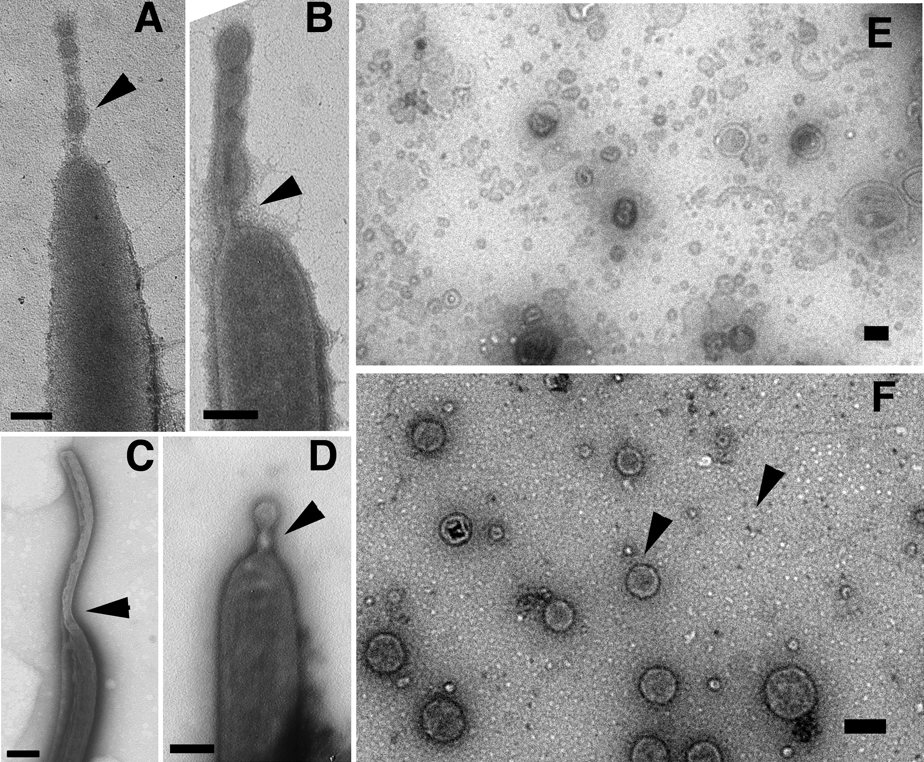

Supplement: Figure S1 — Vesicles released from the tips of fully-grown flagella (A–D) can be purified from the medium (E, F). A, B: Flagellar membrane vesicles (FV) are shed from the tips of Chlamydomonas vegetative and gametic cells. Vesicles (A, arrowhead) release near or to one side of the central microtubule caps on WT flagella, which contain central microtubules, but from the tips D, arrowhead) of pf18 flagella, which lack central microtubules and caps. B, C: Larger FV released with 20 mM Na+-pyrophosphate. The end of the axoneme is indicated by the arrow. D: FV (arrows) releasing from pf18 flagella that lack central microtubules. E, F: Shed flagellar vesicles (FV) were purified from the medium in which cells were incubated for 6–8 hours. These vesicles varied in diameter: 78% of vesicles released from WT cells (E) were 20–50 nm in diameter and the rest were larger; 73% of vesicles released from pf18 were 10–30 nm in diameter. Larger vesicles were produced when flagellar disassembly was induced with sodium pyrophosphate (F). Vesicle diameters range from 60–200 nm diameter and have varied shapes. Bars = 200 nm (TIF) [file pone.0053366.s001.tif]

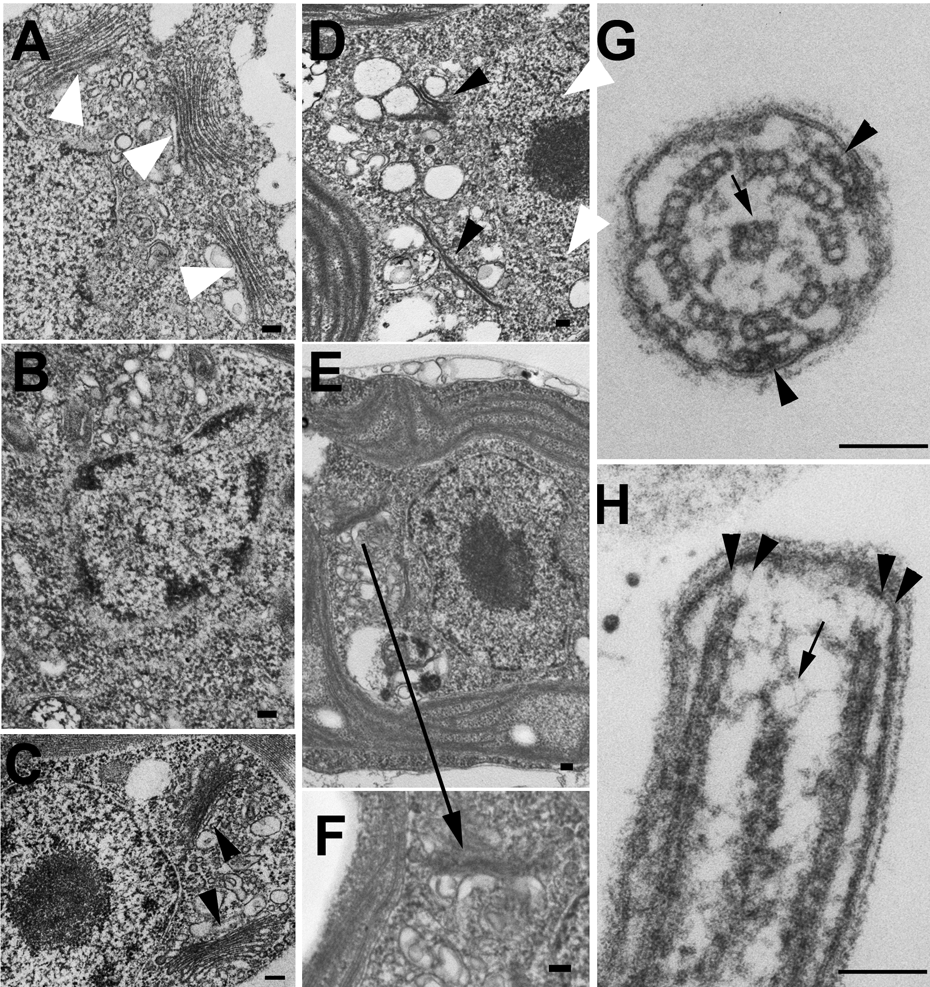

Supplement: Figure S2 — BFA reversibly destroys visible Golgi in Chlamydomonas cells. To confirm that BFA destroyed Golgi, cells were incubated with 0.4–54 µM BFA for 3 hours and then fixed for TEM. Cross-sections of cells that contained the nucleus and basal bodies were analyzed for the presence or absence of Golgi. In 51 sections of control cells, 42 (82%) of cells had one or more Golgi (A). Analysis of 51 sections of cells treated with 36 µM BFA (B) revealed no cells with Golgi; a repeat of this experiment found one possible Golgi in more than 200 images of the cross-sectioned cells. When cells were washed out of BFA and fixed after 30 min., analysis of 58 cross-sections revealed that 79% of the cells had one or more normal-appearing Golgi (C). With lower BFA, normal Golgi stacks were never observed, but possible Golgi remnants were found in cells treated with 1.4 µM (D) or 2.8 µM BFA (E, F). Flagella in BFA-treated cells looked normal and contained both IFT particles (G) and the distal tips of the A tubules attached to the membrane by distal filaments (H). Therefore, Golgi were absent in BFA-treated cells in which flagellar assembly is partially (3.6 µM) or completely (36 µM) inhibited and in which flagella shorten. Bars = 0.1 µm. (TIF) [file pone.0053366.s002.tif]

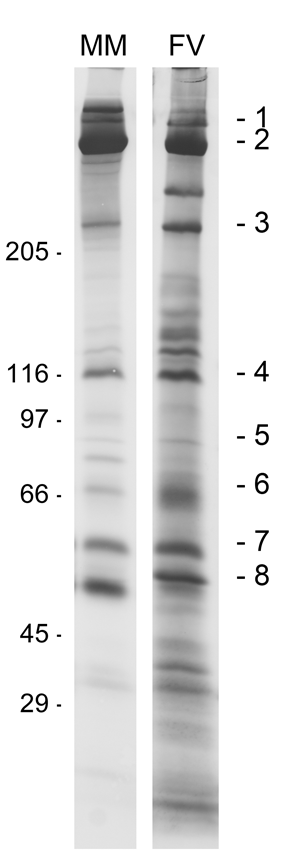

Supplement: Figure S3 — Western blots of detergent-solubilized flagellar proteins (MM) and released flagellar membranes (FV) stained with AP-streptavidin to reveal biotinylated proteins. This is one of 5 gels that were analyzed for data presented in Table 1. (TIF) [file pone.0053366.s003.tif]
